# A Review of Thermoplastic Resin Transfer Molding: Process Modeling and Simulation

**DOI:** 10.3390/polym11101555

**Published:** 2019-09-24

**Authors:** Tatyana Ageyeva, Ilya Sibikin, József Gábor Kovács

**Affiliations:** Department of Polymer Engineering, Faculty of Mechanical Engineering, Budapest University of Technology and Economics, Műegyetem rkp. 3, H-1111 Budapest, Hungary; ageyevat@pt.bme.hu (T.A.); sibikini@pt.bme.hu (I.S.)

**Keywords:** T-RTM, modeling, polymerization kinetics, crystallization kinetics, rheokinetics, in situ polymerization, reactive PA-6

## Abstract

The production and consumption of polymer composites has grown continuously through recent decades and has topped 10 Mt/year. Until very recently, polymer composites almost exclusively had non-recyclable thermoset matrices. The growing amount of plastic, however, inevitably raises the issue of recycling and reuse. Therefore, recyclability has become of paramount importance in the composites industry. As a result, thermoplastics are coming to the forefront. Despite all their advantages, thermoplastics are difficult to use as the matrix of high-performance composites because their high viscosity complicates the impregnation process. A solution could be reactive thermoplastics, such as PA-6, which is synthesized from the ε-caprolactam (ε-CL) monomer via anionic ring opening polymerization (AROP). One of the fastest techniques to process PA-6 into advanced composites is thermoplastic resin transfer molding (T-RTM). Although nowadays T-RTM is close to commercial application, its optimization and control need further research and development, mainly assisted by modeling. This review summarizes recent progress in the modeling of the different aspects of the AROP of ε-CL. It covers the mathematical modeling of reaction kinetics, pressure-volume-temperature behavior, as well as simulation tools and approaches. Based on the research results so far, this review presents the current trends and could even plot the course for future research.

## 1. Introduction

Reactive thermoplastic systems for composite production have recently attracted a great deal of interest from both academia and industry [1,2]. Such systems comprise a mixture of monomers and/or oligomers (in either cyclic or linear form) that are converted into polymers through the addition of an initiator and activator. Due to their low molecular weight, monomers and oligomers exhibit water-like viscosity and can easily impregnate dry reinforcement, thus overcoming the main problem of thermoplastic matrices in composite manufacturing. Reactive thermoplastic systems offer the advantages of thermoplastics such as recyclability, weldability, higher toughness, and overmoldability. Due to their low viscosity, reactive thermoplastics can be used in liquid composite molding (LCM) techniques, such as thermoplastic resin transfer molding (T-RTM) or vacuum infusion. Reactive thermoplastics are polymerized in situ, i.e., in the mold. Currently, a number of reactive thermoplastics suitable for in situ polymerization are available, including thermoplastic polyurethanes and polyesters, polyamides (PAs), polycarbonates, and polymethylmethacrylates [3,4]. The most promising of these polymers is PA-6, as it is a compromise solution from a processing point of view. PA-6 belongs to the family of technologically relevant engineering thermoplastics; more than 5 Mt is produced annually (2015) [5]. Reactive PA-6 can be processed at moderate temperatures (130–180 °C) and converts quickly, usually in the range of 2–60 min depending on the chemical formulation of the reactive system. Reactive PA-6 can be synthesized from cyclic monomer ε-caprolactam (ε-CL) via anionic ring opening polymerization (AROP). Rapid polymer conversion together with the fast impregnation of the reinforcement enable a cycle time of 2–10 min for composite manufacturing, thus satisfying the demands of mass production.

The AROP of CL was first mentioned in the 1940s [6,7], but the development of its chemistry boomed during the 1980s [6,8,9,10]. However, despite the great potential of reactive PA-6 for LCM, the lack of cooperation between polymer chemists and composite processing engineers at that time did not allow thermoplastic LCM to be commercially feasible [3]. A new era of reactive PA-6 composites started in the 2010s with the development of the industrial equipment for thermoplastic LCM. Today, several ready-to-use production lines for different reactive thermoplastic LCM techniques exist [1]. For example, reactive thermoplastic pultrusion lines have been developed independently by academic research groups at the École polytechnique fédérale de Lausanne [11,12,13] and Beijing University of Chemical Technology [14,15], as well as by industrial companies, such as Krauss Maffei (München, Germany) [16] and CQFD Composites (Wittenheim-Alsace, France) [17]. A research group from Delft University investigated vacuum-assisted resin transfer molding with reactive PA-6 for the production of wind turbine blades [18,19,20,21,22,23,24,25,26,27,28,29]. Successful industrial production of a textile-reinforced composite with in situ polymerized CL via T-RTM was demonstrated by KraussMaffei [30,31] and Engel [32]. All these examples prove that reactive PA-6 for LCM is currently close to being ready for industrial application. This article focuses on T-RTM.

**Figure 1:**
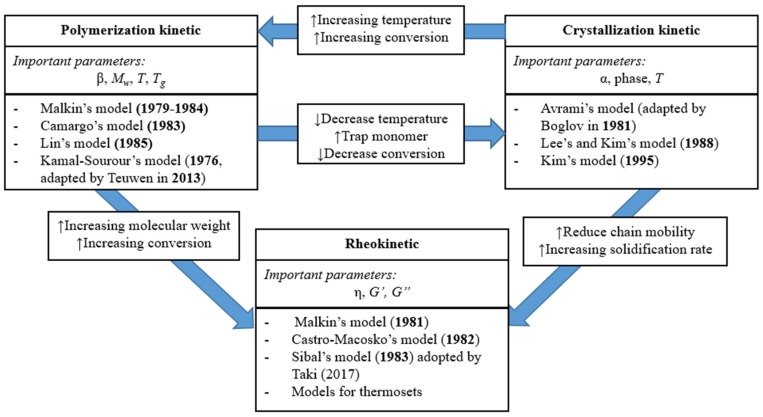
Interaction between polymerization, crystallization, and rheology in the anionic ring opening polymerization (AROP) of caprolactam (α—degree of crystallinity, β—degree of conversion, *M_w_*—molecular weight of the polymer, *T*—processing temperature, *T_g_*—glass transition temperature of the polymer, η—viscosity, *G*’ and *G*’’—the storage modulus and loss modulus, respectively) (based on reference [33], reproduced with copyright permission from Elsevier, 2018).

Even though today the AROP of CL could be considered a well-understood process on the verge of industrial application, optimization and control of the technological process is still unreliable. The process can be optimized either by trial and error or by modeling. Regardless of the fact that a combination of these two approaches is generally used today, process modeling is a promising option. Modeling LCM with the in situ polymerization of CL is associated with certain difficulties, as several mutually dependent physical and chemical processes have to be considered. While flowing through the porous media, the reactive mixture can polymerize and crystallize simultaneously; with the progress of polymer conversion, the viscosity of the reactive mixture increases exponentially and it affects the flow of the resin. Fluid momentum and mass transfer in a continuum liquid media as well as heat transfer play a key role in the process. For the most feasible and accurate reactive thermoplastic LCM model, it is necessary to simulate all the above-mentioned physical and chemical phenomena. The modeling of resin flow, fiber infiltration, and heat transfer have already been well-investigated in the case of thermoset LCM. Moreover, several simulation program packages that directly support thermoset LCM modeling are already available (Ansys, PAM-RTM, and others). The blank spot in the case of T-RTM is the simulation of the chemical reaction coupled with the simulation of the physical phenomena. A number of analytical models for the polymerization, crystallization and rheological behavior of reactive PA-6 have already been developed (Figure 1), and their validity has been proved for certain conditions. However, a modeling system that combines resin flow, infiltration, and in situ polymerization simulation has not been developed yet. The creation of such a modeling system will enable the optimization of thermoplastic LCM without using an excessive amount of time and physical resources. Therefore, it is important to model in situ polymerization for T-RTM.

The aim of the current paper is to summarize the recent progress in the field of T-RTM process modeling and simulation with a particular focus on CL-based systems. The study has the following chapters: T-RTM equipment and process parameters, the modeling of polymerization and crystallization kinetics, the modeling of rheokinetics, the influence of pressure on the reaction rate, pressure-volume-temperature modeling, and the simulation of the T-RTM process.

## 2. T-RTM Equipment and Process Parameters

**Figure 2:**
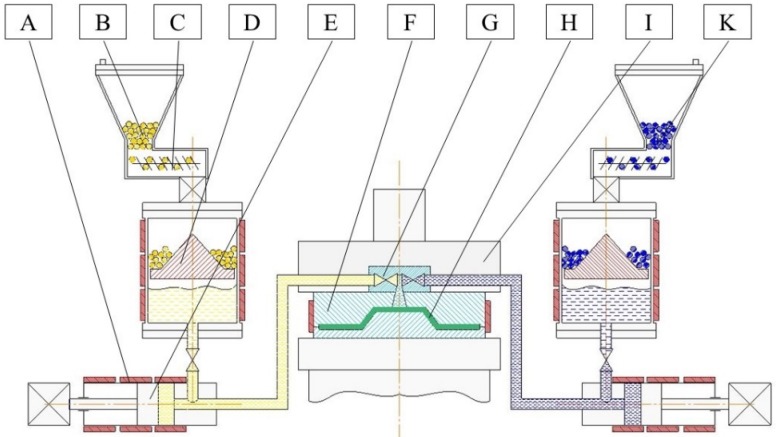
Scheme of the T-RTM process: A: heating; B: ε-caprolactam (ε-CL)+initiator (masterbatch); C: feeding system; D: melting unit; E: feeding pump; F: mold; G: mixing head; H: product; I: clamping unit; K: ε-CL+activator (masterbatch).

A typical production cell for T-RTM consists of a dosing machine, a mold, and a clamping unit (see Figure 2) [1]. The dosing machine serves to melt, pressurize, transport, and mix the reactive components. The mold gives the product its final shape, contains the fiber preform and provides the necessary temperature to initiate the AROP reaction. Conditions in both the dosing unit and the mold are managed through the adjustment of processing parameters. They include injection pressure, injection speed, temperature of the reactive melt in the dosing unit, and the temperature (heat rate) of the mold. In order to be able to model and simulate the T-RTM process, researchers need to understand the influence of these parameters at each processing step.

When the reactive mixture enters the mold, two things happen: fiber infiltration and the in situ chemical reaction itself both occur. The former is well-researched for thermoset resins while the latter needs additional investigation for ε-CL. As the AROP reaction starts, the viscosity of the melt increases and at the level of 1 Pa∙s, further fiber impregnation is impossible. This is why it is critical to understand how matrix viscosity as well as reaction rate changes in time as a function of temperature, injection speed, and pressure. The influence of temperature on the AROP rate is well-researched, while the role of injection pressure and speed is usually ignored and considered only at the fiber infiltration stage. At the same time, it can be assumed that the contribution of injection pressure and injection speed to the chemical reaction rate can be significant.

**Figure 3:**
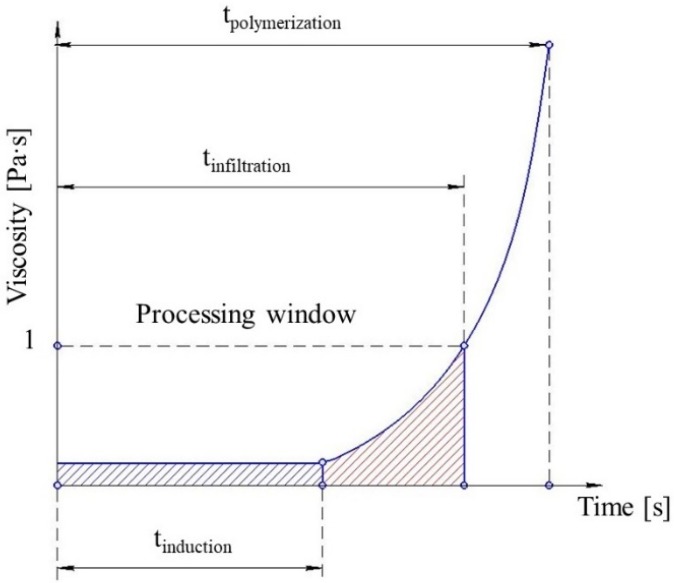
Schematic representation of processing window limitations based on the viscosity of the reactive mixture.

The reaction time of the melt can be divided into the induction period, when no change in viscosity is observed, and the period when the reaction accelerates significantly and viscosity increases (Figure 3). Their duration defines the processing window—the time available before the reactive mixture reaches the viscosity of 1 Pa∙s. The processing window can be estimated either via simulation or by experiment. The simulation helps to reduce the amount of wasted materials and shortens the production start-up period. It also helps to define theoretical production cycle time. The cycle time is one of the main characteristics determining the commercial feasibility of the process. It must be as short as possible and it can be optimized by T-RTM process simulations. The optimum solution would be to have the shortest cycle time with a sufficient processing window for fiber infiltration. Thus, the focus of this review is on how processing parameters influence the AROP reaction rate rather than their influence on the mechanical properties, shrinkage, warpage, and morphology of the product.

## 3. The Kinetic Modeling of Polymerization and Crystallization

A distinct feature of PA-6 is that polymerization and crystallization occur simultaneously [34]. Polymerization and crystallization happening at the same time can significantly influence product quality and the efficiency of the process. Thus, based on references [33,35], early crystallization leads to the trapping of monomers and the reduction of the final conversion, while late crystallization increases the time to demold, thus making the process less feasible. Moreover, late crystallization results in polymer chain branching, thus impairing the ultimate quality of the polymer crystals. Therefore, the prediction of polymerization and crystallization during the AROP of CL is essential for T-RTM process modeling.

The rates of polymerization and crystallization strongly depend on the chemical composition of the reactive mixture; initial polymerization temperature *T*_0_; thermal conditions (isothermal, non-isothermal); and the heating rate [10,36,37]. Further, we will discuss the approaches of modeling polymerization and crystallization kinetics in more detail, as well as highlight the effect of the above-mentioned factors on both processes.

### 3.1. Reaction Chemistry

**Figure 4:**
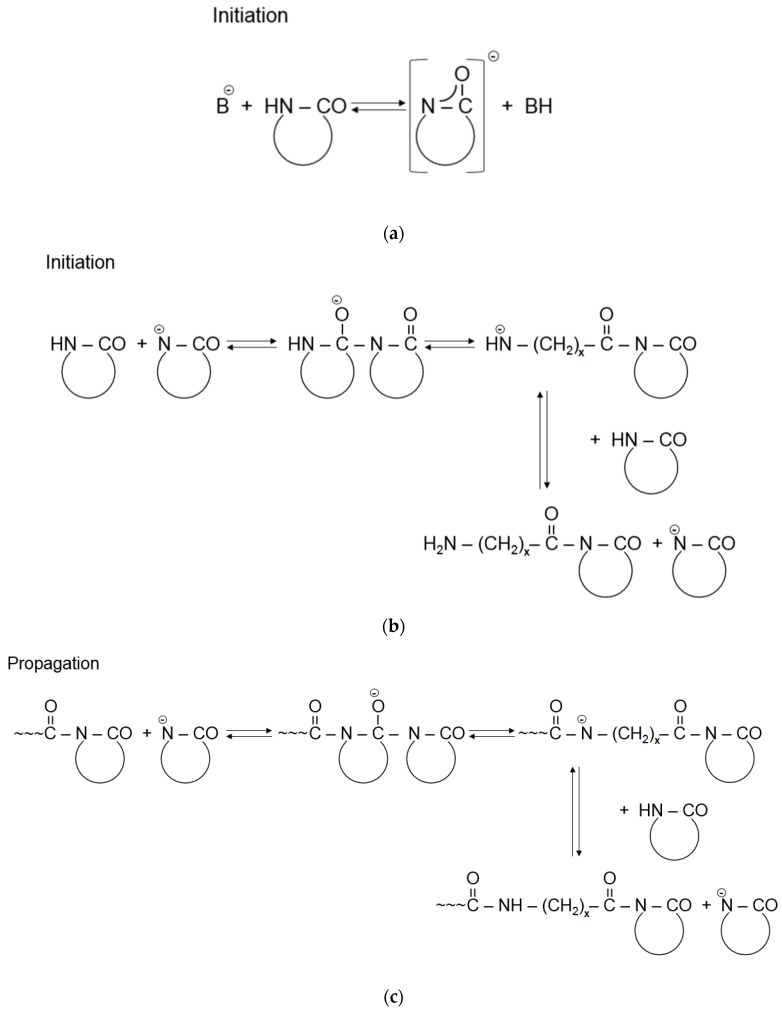
Initiation (**a,b**) and propagation (**c**) of the AROP of ε-CL (Note: x = 5), reprinted from [1] under open access license.

The AROP of ε-CL is a complex chemical reaction consisting of basically two steps—initiation and propagation. The reaction is initiated by strong bases, which form free lactam anions. Initiation includes the opening of the lactam ring and the formation of a primary amine anion, which is more basic than the initial lactam. Therefore, primary amine anions immediately start to react with the surrounding monomers through proton capture, and as a result, a new lactam anion and ω-aminoacyllactam are formed. Propagation includes the repeated nucleophilic attack of the lactam anion and the endocyclic carbonyl group of the non-ionic growth center. Thus, propagation involves the repeated acylation of the lactam anion (Figure 4) [1]. This reaction mechanism is known as a “regular” one but due to the high reactivity of species involved in the AROP series, there are also side reactions [10,38], including deactivating, branching, and crosslinking. These produce side products and irregularities in the resultant polymer chain structure [39].

### 3.2. Polymerization Kinetic Models

The polymerization kinetics of the AROP of CL can be described in two ways—mechanistic and semi-empirical. The mechanistic approach [40,41,42,43,44,45,46,47] takes into account all the side reactions, and due to the complex nature of AROP, it is impractical. The semi-empirical approach lumps all reactions into a single step and describes them in a single Arrhenius-based rate equation, without much consideration of reaction chemistry. Therefore, the semi-empirical approach is mostly used for the kinetic description of AROP. The rate equation is semi-empirical, and its constants should be identified by solving an inverse problem. This procedure implies determining the temperature profile of the reactive mixture experimentally during polymerization. The experimental data obtained are further adjusted with the modeling. Ruso et al. [10,48] and Teuwen [25] wrote a comprehensive review summarizing research in this field. We, in the current study, will describe the most important milestones in the development of kinetic models in order to tell the entire story.

#### 3.2.1. The Malkin Model (1979–1984)

The oldest and most frequently used kinetic model for AROP was developed by Malkin and his research team in the 1980s [49,50,51,52,53]. The macrokinetic description of polymerization they proposed was based on the joint solution of two equations—the polymerization kinetic (1) and thermal conductivity Equations (2):

(1) $$\frac{d\beta }{dt}=Kf\left(\beta \right)\exp\left(-\frac{E}{RT}\right)$$

(2) $$\frac{\partial T}{\partial t}=a\left(\frac{\partial^{2}T}{\partial r^{2}}+\frac{2}{r}\frac{\partial T}{\partial r}\right)+\frac{Q}{c}\frac{\partial \beta }{\partial t}$$

where β is the degree of conversion, *T(r, t)* is a temperature function that varies with the coordinates and time, *K* depends on the catalyst and activator concentrations, f(β) is the kinetic function, *E* is the activation energy of the process, *R* is the universal gas constant, *a* is thermal diffusivity, *r* is a coordinate in a cylindrical coordinate system, *Q* is the thermal effect of the reaction and *C* is heat capacity.

They defined the kinetic constants *K, E, Q*, and f(β), minimizing the difference between the experimental and predicted results. The important conclusion the authors made was that the AROP of CL has an autocatalytic character, which was illustrated in the kinetic function:

(3) $$f\left(\beta \right)=\left(1-\beta \right)\left(1+B_{0}\beta \right)$$

where $B_{0}$ is an autocatalytic factor.

Finally, the autocatalytic rate equation formulated by Malkin:

(4) $$\frac{d\beta }{dt}=A_{0}\left(1-\beta \right)\left(1+B_{0}\beta \right)\exp\left(-\frac{E}{RT}\right),$$

where $A_{0}$ is the constant representing the number of collisions of the molecules.

Later Malkin and co-workers [53] modified the model by taking into account initiator and activator concentrations:

(5) $$\begin{array}{c} \frac{d\beta }{dt}=k\frac{\left[I\right]\left[A\right]}{{\left[M\right]}_{0}}\left(1-\beta \right)\left(1+\frac{m\beta }{{\left(\left[I\right]\left[A\right]\right)}^{1/2}}\right)\exp\left(-\frac{E}{RT}\right),\\ \left[I\right]=f\times \left[A\right], \end{array}$$

where [I] is initiator concentration; [A] is activator concentration; ${\left[M\right]}_{0}$ is initial monomer concentration; *k* is the pre-exponential factor; *m* is the constant characterizing the intensity of the auto-acceleration of the reaction, and *f* is the functionality of the activator.

#### 3.2.2. The Camargo Model (1983)

Malkin assumed the AROP of CL was a first order reaction, which was further modified by Camargo et al. [54]. They introduced the reaction order parameter *n*:

(6) $$\frac{d\beta }{dt}=A_{0}{\left(1-\beta \right)}^{n}\left(1+B_{0}\beta \right)\exp\left(-\frac{E}{RT}\right)$$

#### 3.2.3. The Lin Model (1985)

The next transformation of the kinetic equation was proposed by Lin et al. [44], who considered the autocatalytic factor *B*_0_ to be a function of conversion:

(7) $$\begin{array}{c} B_{0}=\frac{B^{\prime }}{1-B^{\prime }\beta },\\ \frac{d\beta }{dt}=A_{0}{\left(1-\beta \right)}^{n}\left(1+\frac{B^{\prime }}{1-B^{\prime }\beta }\beta \right)\exp\left(-\frac{E}{RT}\right), \end{array}$$

where $B^{\prime }$ is a new autocatalytic function.

Despite the good fitting results, Lin’s model demonstrates less agreement with experimental data than Malkin’s, and is therefore almost abandoned now [48].

#### 3.2.4. The Kamal-Sourour Model (1973–1976) adopted by Teuwen (2011–2013)

Kamal-Sourour’s kinetic equation (8) was initially developed for the curing of epoxy resins [55,56,57]. However, Teuwen [24,25] recently proved that this model could also adequately describe the AROP of CL. This fact hints at the similarities between AROP and the curing of thermosets.

(8) $$\begin{array}{c} \frac{d\beta }{dt}=\left(k_{1}+k_{2}\beta^{m}\right){\left(1-\beta \right)}^{n},\;\\ k_{1}=A_{1}\exp\left(-\frac{E_{a1}}{RT}\right),\\ k_{2}=A_{2}\exp\left(-\frac{E_{a2}}{RT}\right), \end{array}$$

where $k_{1}$ and $k_{2}$ are the rate constants, while *m* and *n* are constants independent of temperature.

Ruso et al. [48] compared various kinetic models and confirmed that Kamal-Sourour’s model fits the AROP of CL best.

### 3.3. AROP Crystallization Kinetic Models

**Figure 5:**
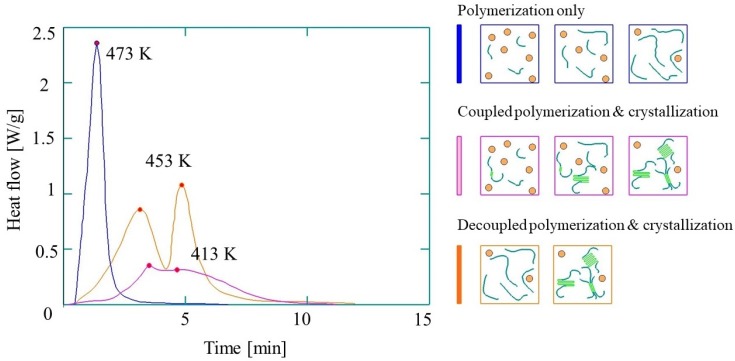
Differential Scanning Calorimetry (DSC) curves of the synthesis of reactive PA-6 (based on reference [58], reproduced with copyright permission from Elsevier, 2017).

A distinct feature of reactive PA-6 is that polymerization and crystallization are concurrent. Depending on the thermal mode (polymerization temperature, heating rates, and cooling rates), polymerization and crystallization can be either coupled or decoupled, or polymerization can take place alone (Figure 5). Therefore, in most cases, crystallization can hardly be considered independent of polymerization, and therefore these phenomena and their modeling cannot be separated.

Both polymerization and crystallization have an exothermic nature. Therefore, their separation from the experimental thermograms is not a trivial task. At the same time, it is very important to develop a model for crystallization and to know crystallization behavior for the shortest cycle time and the optimization of the dimensional stability of the part after demolding. Several analytical crystallization models have been developed for the AROP of CL.

#### 3.3.1. The Avrami Model Adapted by Bolgov (1979–1981)

Bolgov et al. [59,60,61] proposed the standard Avrami equation to describe the crystallization kinetic of the AROP of CL under isothermal conditions:

(9) $$\begin{array}{c} \alpha \left(t\right)=1-\exp{\left[-\frac{t}{t_{0}}\right]}^{n},\\ t_{0}=C\cdot \exp\left[\frac{\psi T_{m}}{T\left(T_{m}-T\right)}\right], \end{array}$$

where α(t) is the degree of crystallinity; *t*_0_ is characteristic crystallization time; *C, n,* ψ are kinetic constants, and $T_{m}$ is the melting temperature of the polymer.

However, the application of the Avrami equation is limited by the isothermal polymerization conditions, can only be used for single-stage (or primary) crystallization, and is not sufficient when secondary crystallization occurs.

#### 3.3.2. The Malkin Model (1984)

Later, Malkin et al. [62,63] proposed an autocatalytic equation to describe the AROP of CL synthesized under isothermal and non-isothermal conditions:

(10) $$\begin{array}{c} \dot{\alpha }=K_{0}\left(\alpha_{eq}-\alpha \right)\left(1+C_{0}\eta \right),\\ K_{0}=\frac{I_{0}W_{0}}{\alpha_{eq}},\\ C_{0}=G_{0}D_{0}{\left(V_{0}I_{o}W_{0}\right)}^{-1}, \end{array}$$

where $K_{0},\;C_{0}$ are constants, and $\alpha_{eq}$ is the equilibrium degree of crystallinity.

#### 3.3.3. The Lee and Kim Model (1988)

Lee and Kim [64] further modified Equation (10), assuming that crystallization involves heterogeneous nucleation, and crystal growth occurs via the formation of three-dimensional spherulites:

(11) $$\begin{array}{c} \frac{d\alpha }{dt}=A\cdot \exp\left(-\frac{E_{D}}{RT}\right)\cdot \exp\left(-\frac{\psi T_{m}}{T\left(T_{m}-T\right)}\right)\cdot \alpha^{\frac{2}{3}}\cdot \left(\alpha_{eq}-\alpha \right),\\ A=C\cdot A_{0}, \end{array}$$

where *A* is a pre-exponential factor, $E_{D}$ is the activation energy for the transformation of the amorphous phase to the crystalline phase, and ψ is a constant.

#### 3.3.4. The Kim Model (1997)

Kim et al. [65] modified the Avrami equation as follows:

(12) $$\alpha \left(t\right)=\alpha_{eq}\cdot \beta \cdot (1-\exp\left(-K{\left(t-\theta \right)}^{n_{c}}\right),$$

where *K* is the rate constant of crystallization; $n_{c}$ is the Avrami exponent, and θ is the crystallization induction period.

#### 3.3.5. The Tonoyan Model (2007)

Tonoyan et al. [66] modified the crystallization kinetic equation derived by Malkin et al. [62,63] by adding the conversion degree as a multiplier to the equilibrium degree of crystallinity, thus taking into account that only a small part of the polymer formed is converted into the crystalline polymer:

(13) $$\dot{\alpha }=K_{0}\left(\beta \cdot \alpha_{eq}-\alpha \right)\left(1+C_{0}\cdot \eta \right).$$

#### 3.3.6. The Johnson-Mehl-Avrami-Kolmogorov (JMAC) Equation Adapted by Vicard (2017)

Vicard et al. [67] used a generalized form of the Avrami equation, which is also called the Johnson-Mehl-Avrami-Kolmogorov (JMAC) equation:

(14) $$\dot{\alpha }\left(t-t_{c,0}\right)=nK\left(T\right)\left(1-\alpha \right)\ln{\left(\frac{1}{1-\alpha }\right)}^{\frac{n-1}{n}},$$

where K is the temperature-dependent kinetic constant, *n* is the Avrami constant representing the type of nucleation/growth, and $t_{c,0}$ is the initiation time of crystallization.

### 3.4. Experimental Methods of the Investigation of the Kinetics of the AROP of CL

**Figure 6:**
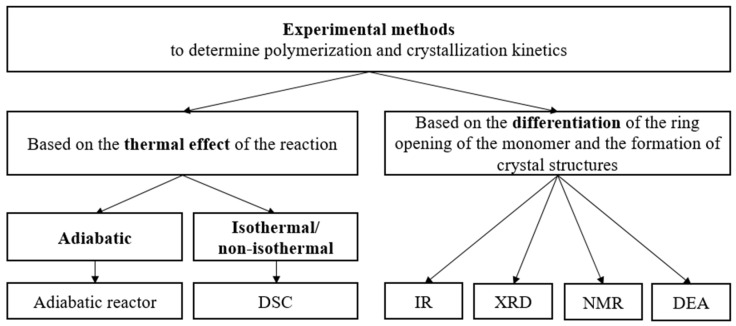
Experimental methods for determining the polymerization and crystallization kinetics of AROP of CL.

The experimental methods for the determination of the kinetic constants of polymerization can be divided into two groups—methods based on the thermal effect of the reaction (adiabatic, isothermal, and non-isothermal), and methods that can differentiate the ring opening of the monomer and the formation of crystal structures (Infrared-Spectroscopy (IR), X-Ray Diffraction (XRD), Nuclear Magnetic Resonance (NMR) and Dielectric Analysis (DEA)) [68] (Figure 6).

#### 3.4.1. Adiabatic Reactor Tests

Reactive PA-6 synthesis could be characterized in bulk with an adiabatic reactor [69,70,71,72,73,74]. This approach implies monitoring the temperature of the reactive mixture in the middle of a thermally insulated vessel during polymerization and crystallization. A certain advantage of this method is the comparatively large amount of the reactive mixture (100–500 mL), which ensures the homogeneity of the material. Temperature change over time is usually measured with thermocouples. However, due to the exothermic nature of both phenomena, their contribution to temperature rise can only be decoupled with complex kinetic models.

#### 3.4.2. DSC Tests

DSC is a well-established experimental technique for the investigation of the polymerization and crystallization of CL on small scale [36,37,42,53,58,61,68,75,76,77,78,79,80]. Typical DSC tests yield data about the heat of the reaction vs. time. Such thermograms provide the qualitative and quantitative interpretation of polymerization and crystallization. Thus, the sequence of polymerization and crystallization can be obtained as well as the degree of their interaction. Khodabakhshi et al. [78] demonstrated a good agreement between small-scale (4–24 mg samples) and bulk polymerization of CL, proving that the polymerization mechanism of CL in a small DSC pan does not differ significantly from that in bulk.

Despite of viability of DSC tests for the investigation of the kinetics of the AROP of CL, the separation of polymerization and crystallization is quite a complicated procedure. Below are different separation methods proposed by different research groups.

#### 3.4.3. The Extrapolation Technique

The first method of separating polymerization and crystallization enthalpies proposed by Malkin et al. [53] involves the determination of kinetic process constants at higher temperatures, where the polymerization and crystallization peaks are clearly distinguished. Then, the obtained constants are extrapolated to the lower temperature regions, where the processes are superimposed. Malkin et al. [53,61] proposed the law of the summation of enthalpies:

(15) $$\dot{q}\left(t\right)=Q_{1}\cdot \dot{\beta }\left(t\right)+Q_{2}\cdot \dot{\alpha }\left(t\right)\cdot \beta ,$$

where $Q_{1}$ and $Q_{2}$ are the overall enthalpies of polymerization and crystallization, respectively.

Equation (15) considers that only the polymerized portion of the material crystallizes.

#### 3.4.4. The Curve-Resolving Technique

The polymerization and crystallization enthalpies from DSC curves could be resolved into their Gaussian components. Karger-Kocsis and Kiss [75] proposed and successfully implemented this technique to separate and quantify the superimposed polymerization and crystallization in the AROP of CL. Taki et al. [80] used this method and applied asymmetric Gaussian function fitting.The achievements in the investigation of the kinetics of the AROP of CL via DSC are summarized in Table 1.

## 4. Rheokinetic Modeling

The viscosity of reactive PA-6 has a significant influence on the impregnation process during LCM. Thus, the viscosity–temperature curve is useful for determining the infusion window, as well as predicting the infusion time at various temperatures. The problem is that the viscosity of reactive thermoplastics changes in a complex way. The viscosity of the monomer CL decreases as the temperature rises (Equation (16)):

(16) $$\eta_{0}\left(T\right)=N\cdot \exp\left(\frac{E_{\eta }}{RT}\right)$$

where $\eta_{0}$ is the viscosity of the monomer at temperature *T*, and $N\;\mathrm{and}\;E_{\eta }$ are material constants.

**Figure 7:**
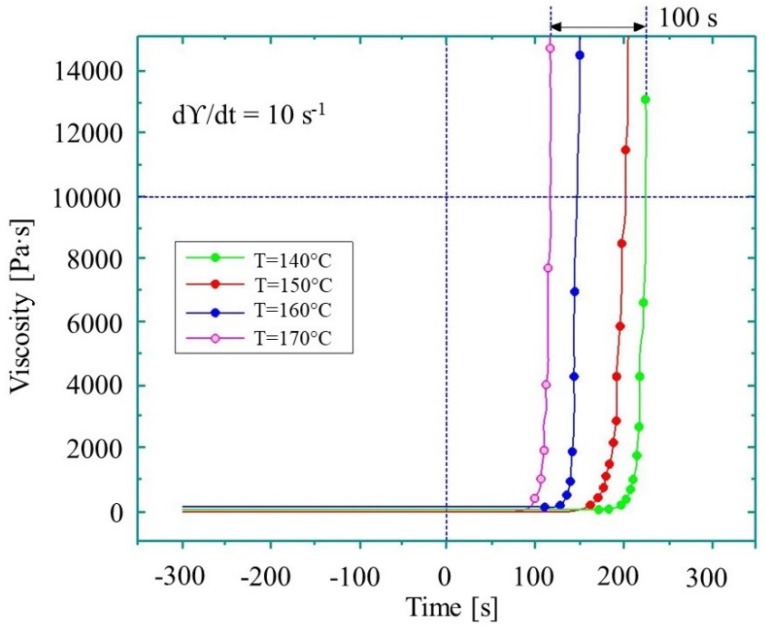
Viscosity of the reactive thermoplastic mixture during the AROP of CL (based on reference [81], reproduced under open access license.).

When the monomer starts to react with the initiator and activator, polymer chains begin to form. At high temperatures, the reaction has an autocatalytic character, thus the viscosity of the material increases exponentially. A typical graph of viscosity change during the AROP of CL is schematically illustrated in Figure 7. Below are the most important models.

### 4.1. The Malkin Model (1981)

Malkin et al. [51] assumed that the reaction system is a solution of the polymer forming in its own monomer. Based on this, they assumed that the viscosity of the reaction mixture depends on the degree of conversion, and could be expressed for the initial stage of AROP in a general form:

(17) $$\eta =K\beta^{a+b},$$

where η is the kinematic viscosity of reaction mixture, *K* is a constant uniting various rheological and kinetic constants, *a* is the constant for the ratio of the viscosity of the solution and molecular weight, and *b* is the constant for the ratio of the viscosity of the solution and polymer concentration.

Malkin and co-workers [51] demonstrated that due to the autocatalytic nature of the AROP of CL, Equation (17) could be transformed as follows:

(18) $$\begin{array}{c} \ln\;\eta =A+\left(a+b\right)\left(c_{0}-m\right)k_{0}t\cdot \exp\left(-\frac{U}{RT_{0}}\right),\\ m=\frac{E∆}{RT^{2}},\\ A=\ln K-\left(a+b\right)\ln c_{0}, \end{array}$$

where *K* is a constant uniting various rheological and kinetic constants, *c*_0_ is an autocatalytic constant, ∆ is the total rise in temperature due to the exothermic effect, $k_{0}$ is the rate constant of the reaction, and *t* is time.

The model can be used for the initial stages of the reaction. The rate equation gives the best results when *a + b* = 12.

### 4.2. The Sibal Model (1983)

Sibal et al. [82] described the viscosity of the monomer and its dependence on temperature with the Arrhenius equation:

(19) $$\begin{array}{c} \eta =\eta_{0}\cdot \exp\left(k\cdot X\right),\\ \eta_{0}=2.7\cdot 10^{-7}\cdot \exp\left(\frac{3525}{T}\right), \end{array}$$

where $\eta_{0}$ is the viscosity of he monomer, *k* is a constant, and the authors called *X* fractional conversion.

Sibal and co-workers found that below 70% conversion, the viscosity rise could be described with the following equation:

(20) $$\eta =\eta_{0}\cdot \exp\left(k_{\eta }\beta \right),$$

where $k_{\eta }$ is the constant.

**Figure 8:**
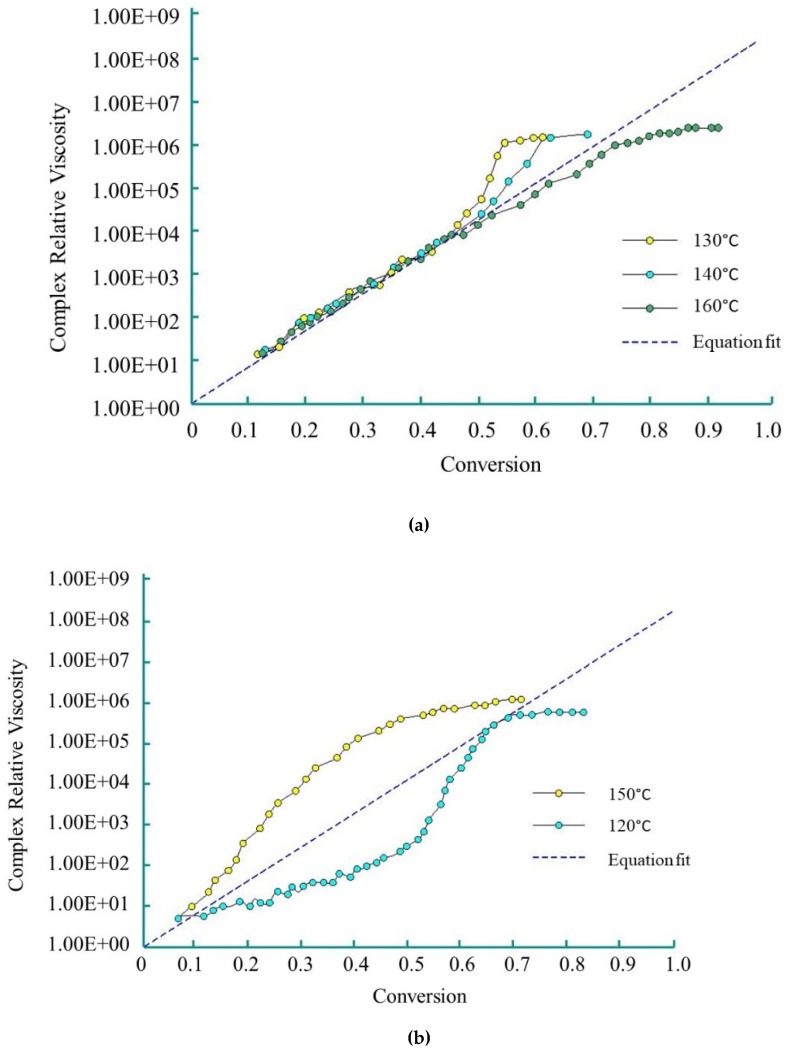
Complex relative viscosity (*η/η*_0_) vs. conversion for polymerization (based on references [83], reproduced with copyright permission from Elsevier, 1997): (**a**) at 120 °C, 140 °C and 160 °C; (**b**) at 120 °C and 150 °C.

Dave et al. [83,84] used the Sibal model to define the complex growth of viscosity during the AROP of CL. The authors used CLMgBr as an initiator (133 mol L^−1^), and isophthaloyl-bis-CL as an activator (90 mol L^−1^). The viscosity change was measured under isothermal conditions (over the range of 120 °C–160 °C) with a rheometric dynamic analyzer. The reactive mixture was delivered to the rheometer platen gap by the injection of two streams consisting of molten CL and initiator, and molten CL and activator through a static mixer. Viscosity was measured during polymerization. Dave and co-workers demonstrated that Sibal’s model shows good agreement with experimental results up to 50% conversion in the temperature range of 130 °C to 160 °C (Figure 8a) for the examined reactive mixture. They describe this phenomenon with the following equation:

(21) $$\frac{\eta }{\eta_{0}}=\exp\left(19.6\cdot \beta \right)\;\mathrm{for}\;\beta <0.5.$$

However, at 150 °C, due to simultaneous polymerization and crystallization, relative viscosity rises non-linearly. The non-linear growth of relative viscosity at 120 °C is caused by ‘sluggish’ polymerization (Figure 8b).

### 4.3. The Castro-Macosko Model (1982) Adopted by Taki (2017)

The Castro-Macosco viscosity model was initially developed for thermosets [85]:

(22) $$\eta =A_{\eta }\cdot \exp\left(\frac{E_{\eta }}{RT}\right)\cdot {\left[\frac{C_{g}^{*}}{C_{g}^{*}-C^{*}}\right]}^{A+BC^{*}},$$

where $C_{g}^{*}$ is the gel point of the material, and $A_{\eta },\;A,\;B,\;C^{*}$ are rheological constants.

Although the Castro-Macosko model was developed for thermoset polymers, it was modified by Taki et al. [80] to describe the AROP of CL:

(23) $$\eta =\eta_{0s}\cdot {\left(\frac{\gamma_{g}-\gamma }{\gamma_{g}}\right)}^{E_{m1}+F_{m1\alpha }}\cdot {\left(\frac{\alpha_{\infty }-\alpha }{\alpha }\right)}^{E_{m2}+F_{m2\alpha }},$$

where $E_{m1},\;E_{m2},\;F_{m1},\;F_{m2}$ are rheological constants, $\eta_{0s}$ is the viscosity of the initial solution, $\gamma_{g}$ and γ are the reaction ratios of the monomer at gelation and at any time up to gelation, and $\alpha_{\infty }$ is the degree of crystallinity at the termination of the crystallization process.

Taki and co-workers used CL with the initiator (GAP-1DA) and activator (GAP-1R) supplied by Nagase ChemteX (Japan). For the above-mentioned reactive system, the modified Castro-Macosko viscosity model showed good agreement with the experimental results at 110 °C, 140 °C and 150 °C, but failed at 120 °C and 130 °C [80]. Consequently, the modified Castro-Macosko model proposed by Taki is not applicable for situations when both crystallization and polymerization increase viscosity.

### 4.4. Rheokinetic Models for Thermosets

Teuwen [25] demonstrated that several rheokinetic models initially developed for thermosets could successfully describe the change of viscosity during the AROP of CL. Among these models are:

- the Stolin-Malkin model [86]:

(24) $$\eta \left(T,\;\beta \right)=\eta_{0}\cdot \exp\left(\frac{E_{\eta }}{RT}+k\beta \right),$$

where $E_{\eta }$ is the activation energy of the viscous fluid, which is assumed to be independent of the degree of curing, and *k* is the rheokinetic constant.

- the model by Dusi et al. [87]:

(25) $$\eta \left(T,\;\beta \right)=\eta_{0}\cdot \exp\left(\frac{E_{\eta }}{RT}+k\beta^{2}\right).$$

- the Williams-Landel-Ferry model [88]:

(26) $$\ln\frac{\eta }{\eta_{T_{ref}}}=\frac{-C_{1}\left(T-T_{ref}\right)}{C_{2}+T-T_{ref}},$$

where $C_{1}\;and\;C_{2}$ are material system constants and $T_{ref}$ is the reference temperature.

Although in study [25] it was proved that models (24)–(26) describe the rheology during the AROP of CL sufficiently (with a correlation of more than 96% between the equations and the experiments), the authors chose the Sibal model due to its simplicity and high correlation (96.4%).

### 4.5. Experimental Methods for the Rheokinetic Investigation of the AROP of CL

Experimental investigations of the AROP of CL involve standard rheometers. The distinct feature of such measurements is that viscosity is measured during polymerization. However, due to the high reactivity of the reactive mixture, it is sometimes tricky to deliver the samples to the rheometer platens. The recent advances in such measurements are summarized in Table 2.

## 5. The Influence of Pressure on Reaction Rate and Pressure-Volume-Temperature Modeling

Temperature influence on the AROP reaction kinetics of CL cannot be underestimated. However, it is just one of the process parameters which is varied in T-RTM. For example, the influence of pressure on the AROP of CL has not been researched, although studies indicate that it has a certain influence on the reaction rate of thermoset polymers [91,92,93]. This influence is attributed to two factors. First, the reaction accelerates because the material becomes denser and because of thermodynamic phenomena. This is followed by deceleration, when the reaction becomes diffusion-controlled [94]. Since pressure values during T-RTM can vary from 5 to 200 bars (depending on fiber content), and because AROP has certain similarities with the curing of thermosets [1], understanding the influence of pressure is important and should be considered in modeling.

### 5.1. The Behavior of Thermosets under Pressure

Mondragon et al. [91] obtained an expression which combines the effect of both temperature and pressure on the reaction rate constant for epoxy systems:

(27) $$\begin{array}{c} K=K_{0}\cdot \exp\left(-\frac{E}{RT}\right)\cdot \exp\left(\left(-\frac{∆\nu^{*}}{RT}+\frac{1}{V}\frac{\partial V}{\partial P}\right)\cdot \left(P-P_{0}\right)\right),\\ \frac{1}{V}\frac{\partial V}{\partial P}=f\left(P\right)=a+bP, \end{array}$$

where *K* and *K*_0_ are the reaction rate constants at pressure *P* and reference pressure *P*_0_ respectively; *T* is the processing temperature; $∆\nu^{*}$ is the activation volume for the reaction; $\frac{1}{V}\frac{\partial V}{\partial P}$ is the compressibility factor, and *a* and *b* are adjustable parameters.

**Figure 9:**
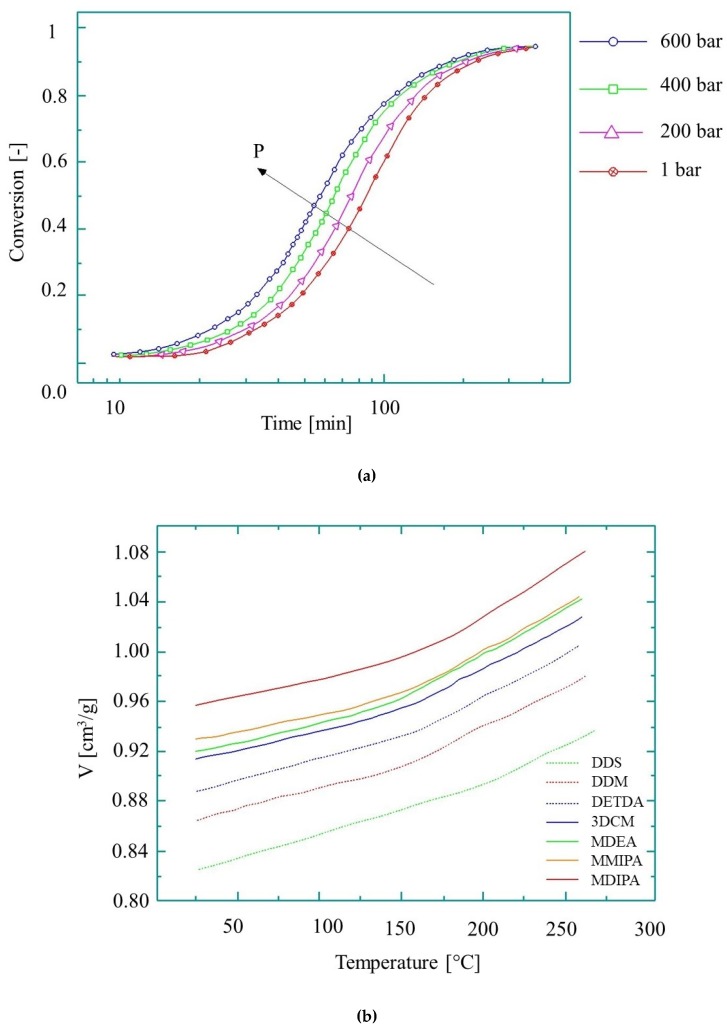
The behavior of epoxy during curing: (**a**) conversion at 160 °C at different pressures [91], reproduced with copyright permission from Elsevier, 2005; (**b**) the pvT diagram (based on reference [92], reproduced with copyright permission from Elsevier, 2004).

Although temperature has the greatest effect on the growth of kinetic rate, pressure-induced growth is also worth examining. As shown in [91], a pressure increase from 200 bar to 600 bar at 160 °C causes the reaction rate constant to increase by 38%, while a temperature rise from 140 °C to 180 °C increases it by 82% (Figure 9a). The cumulative effect of both temperature and pressure on the reaction rate, however, will be different from the individual effects of the parameters, and therefore this needs to be investigated.

Another issue for T-RTM process modeling is the pressure-volume-temperature (pvT) behavior of reactive PA during its transformations (polymerization and crystallization). Specific volume is a fundamental parameter to determine shrinkage and residual stresses in the polymer. Therefore, understanding pvT behavior is essential for process modeling. The pvT diagrams for the curing of epoxy are shown in Figure 9b [92]. The pvT behavior of thermoset resins can be described with the state equation [95]. It is only valid for *X* < *X_gel_* and not valid in the glassy state or during the transformation:

(28) $$\frac{dV}{V}=\frac{1}{V}{\left(\frac{\partial V}{\partial T}\right)}_{P,X}dT+\frac{1}{V}{\left(\frac{\partial V}{\partial X}\right)}_{P,T}dX+\frac{1}{V}{\left(\frac{\partial V}{\partial P}\right)}_{T,X},$$

where *X* – is the relative degree of conversion.

The AROP of CL typically takes place at a temperature lower than the melting temperature of PA-6 and therefore we assume that during the polymerization stage, the pvT nature of the reactive mixture is close to that of thermosets. However, PA-6 is a semicrystalline polymer and during subsequent cooling, it would most likely demonstrate thermoplastic behavior.

### 5.2. Thermoplastic Behavior under Pressure

**Figure 10:**
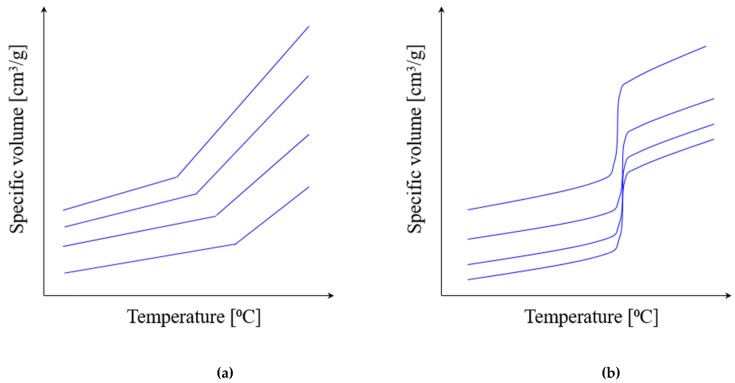
The typical pvT diagrams for thermoplastic polymers (schematically): (**a**) amorphous thermoplastics; (**b**) semi-crystalline thermoplastics.

Typical pvT diagrams have a different shape for amorphous and semi-crystalline thermoplastics (Figure 10).

### 5.3. Thermoplastic Behavior under Pressure

Typical pvT diagrams are different for amorphous and semi-crystalline thermoplastics (Figure 10).

The pvT behavior of thermoplastics is most often described with the empirical 2-domain Tait equation [96]:

(29) $$\begin{array}{c} V\left(T,P\right)=V\left(0,T\right)\cdot \left[1-C\cdot \ln\left(1+\frac{P}{B\left(T\right)}\right)\right]+V_{t\;}\left(T,P\right),\\ V\left(0,T\right)=V_{0}\exp\left(\alpha T\right),\\ B\left(T\right)=B_{0}\exp\left(-B_{1}T\right), \end{array}$$

where *V*(*T,P*) is specific volume at temperature *T* and pressure *P*, V(0,T) is specific volume at zero gage pressure, *C* is 0.0894 (universal constant), *B* represents the pressure sensitivity of the material, α is the thermal expansion coefficient, and $V_{t}\left(T,P\right)$ is an additional transition function required for non-amorphous (semi-crystalline) materials.

We assume that the pvT nature of reactive PA-6 has the characteristics of both thermoset and thermoplastic behavior.

### 5.4. Experimental Methods for Measuring pvT Behavior

The piston-die and confining fluid procedures are two standard practical methods to determine the pvT behavior of plastics. The piston-die method involves placing a sample into a rigid, electrically heated cylindrical chamber with a stationary piston at the bottom and a movable piston at the top, which exerts pressure on the sample. The change in specific volume is calculated from the movement of the piston. Although the measuring equipment is simple and specific volume is measured directly, the technique has a weak point: the non-hydrostatic pressure that causes friction on the wall of the measuring chamber and therefore reduces accuracy. The confining fluid technique is free from the above-mentioned drawback. The sample is placed into a rigid chamber covered with a flexible membrane, and surrounded with mercury or silicone oil. Pressure is exerted on the surrounding liquid, and the liquid transmits the pressure to the sample. Therefore, the pressure is purely hydrostatic. However, this method also has a disadvantage: the possible chemical reaction between the sample and the fluid, as well as the indirect measurement of specific volume. Also, both methods are limited by the maximum achievable cooling rate, which is far below the cooling rate during injection molding. Szabo and Kovacs [97,98] developed a measurement procedure, which enables the measurement of the pvT properties of amorphous thermoplastics during injection molding, directly in an injection unit. The method works well for ABS, PS, SAN and PC in the pressure range of 7 to 28 MPa. This method, however, is not yet been adopted for semi-crystalline thermoplastics.

## 6. Simulation of the T-RTM Process

**Figure 11:**
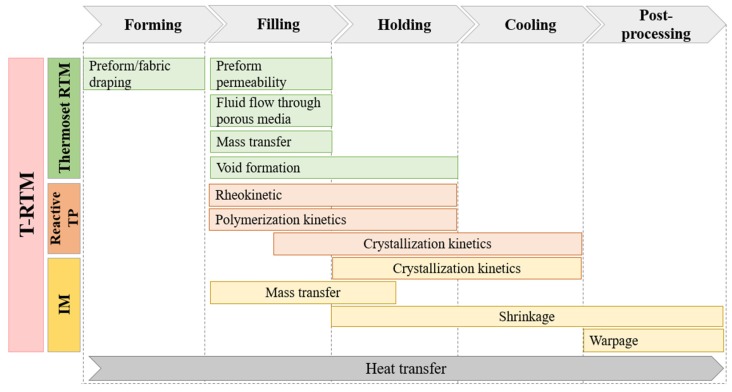
A general concept of T-RTM process simulation as a combination of certain features of thermoset RTM, reactive TP processes, and IM models (RTM—resin transfer molding; TP—thermoplastic; IM—injection molding; T-RTM—thermoplastic resin transfer molding).

Since T-RTM has certain similarities to thermoset RTM, reactive thermoplastic processing and conventional injection molding (IM), it can be considered a hybrid process. Basically, five stages can be distinguished during the T-RTM process: forming the reinforcement, filling the mold, keeping it in a certain thermal mode, cooling and post-processing (Figure 11). Physical phenomena occurring during the forming, filling and holding stages of T-RTM are almost identical to those for thermoset RTM, while the chemical reaction is similar to those occurring in reactive thermoplastic processes. In the cooling and post-processing stages, T-RTM can be described well with processes typical for IM. Consequently, the simulation of T-RTM should be a synergy of approaches and instruments used for RTM, reactive thermoplastic and IM simulations.

Simulation of the T-RTM process is a complex multidisciplinary task that involves several mechanical, physical, and chemical phenomena. Therefore, several sub-models are needed for the development of a general approach (Figure 11). Most studies in this field can be divided into three groups. The first includes simulation of the mold-filling stage. It can predict the location of the flow front, optimize the flow rate, and mold and inlet temperatures, gate locations and optimize process cycle time based on filling time information. Simulation of the filling stage of RTM is extensively studied nowadays and is therefore beyond the scope of this review. The second group includes simulation of polymerization and crystallization. As we demonstrated in the previous sections, robust mathematical and experimental backgrounds have been developed for modeling the chemical reaction. Nevertheless, the merging of polymerization, crystallization, and rheokinetic models with numerical simulations of mold filling is a relatively new topic of research (5–10 years). The third group includes shrinkage and warpage simulations and is also beyond the scope of the current study.

Nowadays, few simulation software products specially developed for RTM and IM process modeling are commercially available. Among them are RTM-Worx (Polyworx), PAM-RTM^TM^ (ESI Group^TM^), and MoldFlow^TM^ (Autodesk^TM^). All of them are able to simulate resin injection; RTM-Workx^TM^ and PAM-RTM^TM^ additionally take into account the deformation of preforms and resin flow based on Darcy’s law. MoldFlow^TM^ and PAM-RTM^TM^ can simulate thermoset curing based on the Kamal-Sourour model and take into account viscosity changes. Additionally, most commercial simulators have the ability to call external user-written subroutines [99], thus allowing the use of the desired polymerization, crystallization and rheokinetic models. There is also free computational fluid dynamic (CFD) software, OpenFOAM^TM^ (ESI Group^TM^), which is able to simulate complex fluid flows involving chemical reactions, turbulence, and heat transfer.

Few recent studies focus on coupling thermo-chemo-mechanical simulations with the flow simulation of reactive thermoset [100,101,102,103,104] and thermoplastic [99,105,106] RTM processes. Imbert et al. [102,103] modeled the RTM process considering the thermo-chemo-mechanical aspects in the flow of reactive thermoset resin mixed on-line in a dual-scale porous medium. The simulations were performed in PAM-RTM^TM^ software. The variables were Darcy’s velocity of the fluid, fluid fraction in a macroscopic element, degree of cure, temperature, and fluid viscosity. The numerical strategy was borrowed from reference [101], and it involved the determination of the pressure field with fluid velocity defined in the elements. The transported quantities (*T,* β, η) were updated at each computational time step. The model was in-plane (two-dimensional), the density and heat capacity of the materials were assumed constants, and the exothermic effect of the reaction was ignored. As a result, the authors performed simulations capable of predicting filling time until the complete saturation of the mold cavity, taking into account temperature, viscosity, and curing progress.

**Figure 12:**
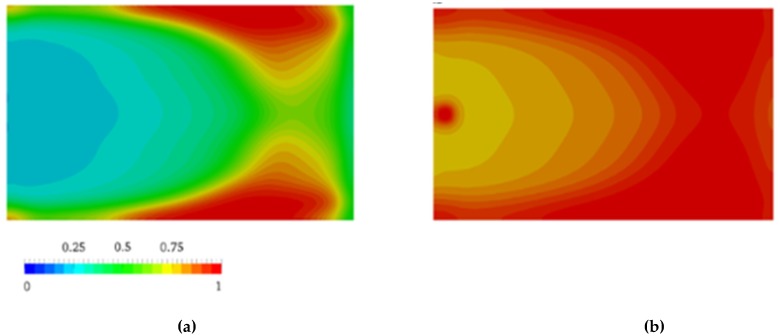
the distribution of polymer conversion β at: (**a**) *t* = 20.0 s; (**b**) *t* = 40.0 s. [105], reproduced with copyright permission from Elsevier, 2014.

Nagy et al. [105] pointed out that flow during the mold-filling phase changes the progress of the AROP of ε-caprolactam dramatically. Therefore, they investigated its influence. The authors combined the Malkin polymerization kinetic model with the CFD software. They ignored the viscosity changes and crystallization kinetics. Inhomogeneities, generated by polymer conversion and governed by the flow, influenced the further course of the reaction and were found to be significant for the entire process. Thus, conversion finished first at the side walls near the outlet of the cavity (Figure 12), which is due to the longer travel distance of the reactive mixture. Neglecting polymerization during the filling phase leads to an overestimation of the processing window (102 s vs. 70 s). Based on the results, the authors considered that the filling phase is a key element during the reactive processing of PA-6.

In another study of theirs [106], Nagy et al. examined the influence of fiber orientation and mold geometry on flow phenomena and reaction progress during the mold-filling phase. The CFD model of flow through the porous media was coupled with Malkin’s kinetic and viscosity models. The authors concluded that while both fiber orientation and mold geometry change the distribution of the degree of polymer conversion, the effect of the latter is more significant.

## 7. Conclusions and Future Prospects

This review summarizes research on T-RTM process modeling and simulation. Based on these studies, we can formulate several key messages:

A robust mathematical background has been developed for the polymerization, crystallization, and rheokinetic modeling of the AROP of ε-CL. The fact that the polymerization of ε-CL can be adequately described by the Kamal-Sourour equation, initially developed for the curing of thermosets, points to some similarities between in situ polymerization and the curing of thermosets. Basically, in both cases, viscosity and the degree of conversion increase as a function of time and temperature.

All reaction kinetic models consider temperature and time as the main parameter that influences the kinetic rate of the reaction. However, none of the models consider the influence of pressure. Although pressure has a certain effect on the curing rate of epoxies, its influence is not as significant as the influence of temperature. High pressure causes the reactive mixture to become denser, draws together reaction groups and as a consequence, accelerates the reaction. We assume that similar phenomena may appear during the AROP of CL, as operating pressures in T-RTM can reach a few hundred bars.

There are a few simulation program packages for RTM process modeling. However, none of them perfectly fulfill the requirements of the T-RTM process. The challenge in the case of T-RTM is modeling in situ polymerization itself. There is no modeling system yet that combines resin flow, fiber infiltration, and the simulation of the AROP reaction. The creation of such a modeling system will enable the optimization of the T-RTM process without using a lot of time or physical resources.

## Figures and Tables

**Table 1:** DSC test results.

Year	The Goal of the Experiments	Monomer/Initiator/Activator (amount)	Thermal Mode	Results/Comments	Reference
1975	A kinetic study on the AROP of CL by DSC in isothermal conditions and in a conversion range of 20–90%.	CL/NaCL/HMDI*	*T_p_* = 180–201 °C	The authors proved that DSC could be effectively used for the investigation of the kinetics of the AROP of CL, and to obtain data in agreement with those for adiabatic measurements.	[42]
1979	The study describes an attempt to separate the polymerization and crystallization in the AROP of CL and the evaluation of the individual enthalpies of each phenomena by means of the curve-resolving technique.	CL/LiL/*N*-acetylcaprolactam (1 mol /%/1 mol %) CL/NaCL/*N*-acetylcaprolactam (1 mol /%/1 mol %) CL/KL/*N*-acetylcaprolactam (1 mol %/1 mol %)	$\frac{dT}{dt}=1.5$ °C/min	$∆H_{p}^{CL}$ = −144 ± 6 J/g $∆H_{c}^{PA-6}$ = −222 ± 5 J/g (100% crystallinity) The curve-resolving technique is proposed.	[75]
1982	An approach to separate crystallization and polymerization enthalpies was proposed. The method is based on the assumption that only the polymerized portion of the reaction mass can be crystallized.	CL/NaCL/AcCL	*T_p_* = 160 °C–200 °C	The autocatalytic nature of the AROP of CL was confirmed. The values of the polymerization and crystallization kinetics constants were determined.	[53]
1992	The study delineates the separation of polymerization and crystallization and the effects of heating rate, catalyst and activator concentration on the kinetics of both processes.	CL/NaH/ABC (1:1; 2:1; 3:1; 5:1)	*m_s_* = 25–40 mg $\frac{dT}{dt}=1.25,\;3,\;5,\;10\;$ and 25 °C/min Cooling rate: 1.25, 3, 5, 10 and 25 °C/min *T_p_* = 150 °C–200 °C	The authors examined the effect of initiator and activator concentration on polymerization and crystallization. They observed polymerization followed by crystallization when polymerization temperature was lower than crystallization temperatures. Crystallization was not observed for polymerization at temperatures higher than the melt crystallization temperature. The driving force for immediate crystallization following polymerization was attributed to the high degree of supercooling.	[36]
2012	The authors discussed the effect of processing parameters, such as polymerization temperature and different initiator/activator concentrations, on the kinetics of polymerization.	CL/C1/C20	-	A temperature of 150 °C and formulation CL/C1/C20 (100/4/4) were demonstrated to be optimal. At 150 °C, AROP led to a similar degree of conversion as in the case of melt-processed PA-6.	[79]
2013	To study the influence of heating strategy on the AROP of CL.	CL/EtMgBr/C_8_H_13_NO_2_ (2.5 mol %/2.5 mol %) CL/C10/C20 (2.5 mol %/1.25 mol %)	$\frac{dT}{dt}=5-20$ °C/min (5 °C/min interval) *T_p_* = 110–150 °C (5 °C interval) *m_s_* = 4–24 mg	The authors proved that the mechanism of polymerization does not differ significantly for small-scale and bulk samples. The relationship between heating rate and polymerization-crystallization was found. The peak temperature of polymerization increases with increasing heating rate. At fast cooling rates, PA-6 quenched before the crystallization process is completed. Increasing the heating and cooling rates result in production irregularities and poorer crystalline structure. Monomer conversion increases when the maximum temperature is increased from 140 °C to 180 °C, and decreases afterwards.	[78]
2017	To determine a heat flow curve, which involved the kinetics of polymerization and crystallization from 50 °C to 250 °C at various heating rates	CL/GAP-1DA/GAP-1R	$\frac{dT}{dt}=1,\;2,\;3,\;4,\;5,\;6$ °C/min T = 30–260 °C	The DSC heat flow curve was separated into polymerization and crystallization curves with the use of the Kamal model and the generalized Avrami model, respectively.	[80]
2017	To characterize the AROP of CL under isothermal and non-isothermal conditions via DSC.	CL/C1/C20P (1.4 mol/kg/2.0 mol/kg)		Polymerization and crystallization have opposite temperature dependencies. The reduction of the temperature of synthesis or heating rate slows down the kinetics of polymerization, while increasing crystallization. Crystallization kinetics strongly depend on the kinetics of chain extension and polymerization controls the overall time of synthesis.	[58]

**Table 2:** Recent advances in the experimental investigation of the rheokinetic behavior of CL during AROP.

Year	Experimental Setup Description	Monomer/Initiator/Activator (Amount)	Thermal Modes	Results/Comments	Reference
1997	*Equipment:* Rheometrics Dynamic Mechanical Analyzer, RMS-800. *Rheometer platens*: disposable aluminum parallel plates (D50 mm, 0.5 mm gap). *The reactive mixture* is delivered into the rheometer platen gap by simultaneous injection of two streams (one containing CL with the initiator, and the other containing CL with the activator) through a static mixture. *Shear mode:* sinusoidal oscillatory shear rate ω=100 rad s^−1^. *Sample surface to volume ratio:* 40 cm^−1^	CL/CLMgBr/acyllactam (133 mmol L^−1^/90 mmol L^−1^)	Isothermal *T_p_* = 120 °C − 160 °C (step 10 °C)	1. The reaction time (required for the complex viscosity level of 10^3^ Pa∙s) for the examined reactive mixture was extremely short: 90 s at 120 °C 45 s at 160 °C. 2. Below 50% conversion, complex viscosity can be described by the Sibal model: ηη0=exp(19.6·β)	[83,84]
2013	*Equipment:* strain-controlled rheometer ARES. *Rheometer platens*: cone and plate (D40 mm, 0.06 mm gap). *The reactive mixture* is premixed and quickly introduced with a syringe into the gap between the preheated cone and plate.	CL/C1/C20 (100/3/3) CL/C1/C20 (100/4/4)	Isothermal *T_p_* = 150 °C − 220 °C (step 10 °C)	The isoviscosity curves vs. time and temperature were obtained for the AROP of CL.	[79]
2017	*Equipment:* parallel plate rheometer MCR-301 *Rheometer platens*: upper plate – disposable aluminum plate D50 mm and D25 mm; lower plate – aluminum cup D75 mm; 0.5 mm gap. *The reactive mixture* is premixed and quickly poured into the rheometer cup.	CL/ GAP-1DA/ GAP-1R	Isothermal *T_p_* = 80 °C − 170 °C (step 10 °C)	The obtained viscosity data was used to determine constants of the modified Castro-Macosko model.	[80]
2017	*Equipment:* rheometer (ARES). *Rheometer platens*: disposable aluminum cone-plate (D25 mm), specially designed to prevent the evaporation of the reaction mixture. A special oil bath was used as isolator. *The reactive mixture* is introduced in powder form. *Shear rates:* 0.1/1/10/100 s^−1^	CL/C10/C20 (4.5 wt%/ 3.0 wt%)	Isothermal *T_p_* = 140 °C − 170 °C (step 10 °C)	It was found that the shear rate strongly influenced the kinetics of polymerization. The higher the polymerization temperature and shear rate are, the shorter polymerization time becomes. The time to reach the viscosity of 100 Pa∙s is between 75 s and 250 s.	[81]
2018	*Equipment:* Thermo Scientific™ HAAKE™ MARS™ Rheometer coupled with FTIR	CL/C1/C20P (100/3/3) (100/4/4)	Isothermal *T_p_* = 190 °C, 230 °C	A correlation between the dielectric parameters and viscosity change was proposed: $\sigma \left(T\right)\cdot \eta {\left(T\right)}^{m}=const$, σ Is ionic conductivity, *m* is the power factor. Time-Temperature-Transformation diagrams were plotted.	[89,90]

## Abbreviations

ABC	adipol-bis-caprolactam;
AROP	anionic ring opening polymerization;
C_8_H_13_NO_2_	N-acetylcaprolactam;
CFD	computational fluid dynamic;
CL	caprolactam;
DEA	Dielectric Analysis;
DSC	Differential Scanning Calorimetry;
EtMgBr	ethyl magnesium bromide;
HCC	hexamethylene-1,6-bis(carbamidecaprolactam);
HMDI	hexamethylenediisocyanate;
KL	potassium salt of lactam;
LCM	liquid composite molding;
LiL	lithium salt of lactam;
NaH	sodium hydrate;
NMR	Nuclear Magnetic Resonance;
PA	polyamide;
T-RTM	thermoplastic resin transfer molding;
XRD	X-Ray Diffraction;
$\frac{1}{V}\cdot \frac{\partial V}{\partial P}$	compressibility factor;
$\alpha_{\infty }$	the degree of crystallinity at the termination of the crystallization process;
[*A*]	activator concentration;
[*I*]	initiator concentration;
[*M*_0_]	initial monomer concentration;
$∆H_{c}$	crystallization enthalpy;
$∆H_{p}$	polymerization enthalpy;
$∆\nu^{*}$	activation volume for the reaction;
*a*	thermal diffusivity;
*C*	heat capacity;
*E*	activation energy of the process;
*m_s_*	sample mass;
*M_w_*	molecular weight of the polymer;
*P*	pressure;
*P*_0_	reference pressure;
*Q*	thermal effect of the reaction;
*R*	universal gas constant;
*T*	processing temperature;
*t*_0_	characteristic crystallization time;
*T_c_*	crystallization temperature;
*T_m_*	melting temperature of the polymer;
*T_p_*	polymerization temperature;
*T_ref_*	reference temperature;
α	degree of crystallinity;
*α_eq_*	equilibrium degree of crystallinity;
β	conversion degree;
*γ_g_*, *γ*	reaction ratio of the monomer at gelation and at any time up to gelation;
*η*	viscosity;
*η*_0_	viscosity of the monomer;
*θ*	crystallization induction period.
